# Supplementary material for: Human Tissue Analysis of Left Atrial Adipose Tissue and Atrial Fibrillation after Cox Maze Procedure
Source: J Clin Med. 2022 Feb 4;11(3):826. doi: 10.3390/jcm11030826 (PMC8837174; doi:10.3390/jcm11030826)
Supplement: Supplementary file 1 [file jcm-11-00826-s001.zip › jcm-1543620-supplementary.pdf]

Supplementary Table S1. Adipose tissue component portion according to AF type.

	SR maintaining	Recurred	*p*-value
Paroxysmal (n=17)	9.75 ± 11.91 (n=14)	21.67 ± 14.43 (n=3)	0.04
Persistent (n=54)	7.93 ± 9.29 (n=41)	16.96 ± 13.72 (n=13)	0.04
Permanent (n=44)	11.63 ± 12.20 (n=28)	14.50 ± 15.36 (n=16)	0.049
